# CD147 is a Novel Interaction Partner of Integrin αMβ2 Mediating Leukocyte and Platelet Adhesion

**DOI:** 10.3390/biom10040541

**Published:** 2020-04-02

**Authors:** David Heinzmann, Moritz Noethel, Saskia von Ungern-Sternberg, Ioannis Mitroulis, Meinrad Gawaz, Triantafyllos Chavakis, Andreas E. May, Peter Seizer

**Affiliations:** 1 Medizinische Klinik III, Kardiologie und Kreislauferkrankungen, Eberhard-Karls Universität Tübingen, 72076 Tübingen, Germany; 2 Institute for Clinical Chemistry and Laboratory Medicine, University Clinic and Faculty of Medicine Carl-Gustav-Carus, TU Dresden, 01397 Dresden, Germany; 3 Department of Cardiology, Innere Medizin I, Klinikum Memmingen, 87700 Memmingen, Germany

**Keywords:** Mac-1, CD147, leukocytes, platelets, adhesion, integrin αMβ2

## Abstract

Surface receptor-mediated adhesion is a fundamental step in the recruitment of leukocytes and platelets, as well as platelet–leukocyte interactions. The surface receptor CD147 is crucially involved in host defense against self-derived and invading targets, as well as in thrombosis. In the current study, we describe the previously unknown interaction of CD147 with integrin αMβ2 (Mac-1) in this context. Using binding assays, we were able to show a stable interaction of CD147 with Mac-1 in vitro. Leukocytes from Mac-1^−/−^ and CD147^+/−^ mice showed a markedly reduced static adhesion to CD147- and Mac-1-coated surfaces, respectively, compared to wild-type mice. Similarly, we observed reduced rolling and adhesion of monocytes under flow conditions when cells were pre-treated with antibodies against Mac-1 or CD147. Additionally, as assessed by antibody inhibition experiments, CD147 mediated the dynamic adhesion of platelets to Mac-1-coated surfaces. The interaction of CD147 with Mac-1 is a previously undescribed mechanism facilitating the adhesion of leukocytes and platelets.

## 1. Introduction

The recruitment of leukocytes and platelets to activated endothelium as well as platelet-leukocyte interactions are of fundamental significance for innate and adaptive immunity, as well as thrombosis [1]. The surface receptor CD147 (basigin, extracellular matrix-metalloproteinase inducer; EMMPRIN) has been shown to be important in the host defense from self-derived as well as invading targets and is a major factor regulating the expression of matrix metalloproteinases (MMPs). CD147 is a pathophysiologically important multi-ligand receptor of the immunoglobulin superfamily. It is expressed in various tissues and cell types, including leukocytes, endothelial cells, and platelets. A range of different proteins, including cyclophilins, monocarboxylate transporters (MCTs) 1-4, CD43, CD44, CD98, NOD2, galectin-3, γ-catenin, apolipoprotein-D, members of the S100 protein family, and integrins, such as α_3_β_1_ and α_6_β_1_, have been reported to interact with CD147 [2,3,4,5,6].

In the context of thromboinflammation, CD147 acts as a pro-inflammatory and pro-thrombotic receptor, eliciting leukocyte chemotaxis and adhesion, as well as platelet activation and subsequent thrombus formation through the binding of various interaction partners [4,7,8,9,10,11,12]. Notably, its interaction with cyclophilin A (CyPA) contributes significantly to various inflammatory diseases. In the context of cardiovascular diseases, CyPA induces leukocyte chemotaxis and adhesion, and myocardial MMP expression, facilitating subsequent myocardial remodeling. Inhibition of extracellular CyPA significantly decreases platelet activation and thrombus formation as well as the formation of monocyte–platelet aggregates [4,7,8,13,14]. Furthermore, our group identified glycoprotein VI (GPVI) to be an adhesion-mediating partner for CD147 on the platelet surface, which is the first time it has been demonstrated that CD147 plays a direct role in cell adhesive events, apart from mediating adhesion via intracellular signaling, leading to the expression of adhesion molecules [11].

Mac-1 (integrin αMβ2, complement receptor 3, CD11b/CD18) is a well-characterized heterodimeric integrin, mostly found on polymorphonuclear leukocytes [15]. As a member of the β2-integrin family, it is known to be involved in the leukocyte adhesion cascade. After the initial contact of the leukocyte with endothelial cells lining the vessel wall, a complex signaling cascade is initiated, leading to a selectin-dependent rolling motion of the leukocyte [16]. To establish a firm contact on the luminal side of the endothelium necessary for extravasation, chemokine-induced inside-out activation of integrins on the surface is necessary [17]. Especially on neutrophils, Mac-1 plays an important role in adhesion to the endothelium upon activation [18,19,20]. In this context, Mac-1-dependent adhesive interactions enable the cells to crawl on the surface of endothelial cells prior to extravasation [16,21]. Numerous cell surface receptors have been identified to interact with Mac-1 in its high-affinity state, including ICAM1-4, JAM-C, Thy-1, RAGE, DC-SIGN, and CD40L [15,22,23]. A plethora of soluble ligands for Mac-1 have been identified, amongst which are fibrin, fibrinogen, plasminogen, factor Xa, heparin, polysaccharides, ssDNA, and dsRNA [15,24]. Mac-1 can also interact with matrix proteins, including vitronectin, collagen, Cyr61, and fibronectin [15]. Mac-1 is also involved in complement-mediated immune responses by recognizing complement C3-opsonized pathogens as well as immune complexes [25].

In this study, we describe for the first time the interaction of integrin Mac-1 with CD147. We provide evidence that CD147 is a novel and relevant binding partner for Mac-1 on leukocytes and platelets.

## 2. Materials and Methods

### 2.1. Mac-1-CD147 Binding ELISA

Binding between Mac-1 and CD147 was evaluated using a modified enzyme-linked immunosorbent assay (ELISA). In a 96-well plate, wells were coated with recombinant Mac-1 (R&D Systems, Minneapolis, MN, USA) or bovine serum albumin (BSA) as the control. Recombinant CD147 was added in increasing concentrations (0–20 µg/mL) for 1 h. After removing CD147 and gentle washing, the wells were incubated with an anti-CD147 antibody (mouse-anti CD147, Abcam, Cambridge, UK) followed by a biotinylated secondary antibody (polyclonal rabbit anti mouse, Dako, Glostrup, Denmark) and a streptavidin/HRP complex (Life technologies, Carlsbad, CA, USA). The binding was detected using 3,3′,5,5′-tetramethylbenzidine (Serva, Heidelberg, Germany). The reaction was stopped using 1 M H_2_SO_4_. The absorption was measured using an ELISA plate reader at 450 nm with a reference value of 570 nm. Measurements from 6 independent experiments were analyzed.

### 2.2. Murine Leukocyte Isolation and Static Adhesion

Murine leukocytes were isolated from bone marrow. CD11b^−/−^ mice were from Jackson Laboratories, and CD147^+/−^ mice were a kind gift from Professor R. A. Nowak (Urbana, IL, USA). The femur and tibia were removed from sacrificed CD11b^+/+^ and CD11b^−/−^ (hereafter designated Mac-1^+/+^ and Mac-1^−/−^, respectively, n = 5), as well as from CD147^+/−^ and CD147^+/+^ (n = 12, respectively) mice (with a C57Bl/6 background). Throughout this, all efforts were made to minimize animal suffering. The bone marrow was washed out of the bones using RPMI 1640 medium (Life technologies, Carlsbad, CA, USA) supplemented with 10% FCS (Life technologies, Carlsbad, CA, USA), 1% Pen/Strep (Sigma-Aldrich, St. Louis, MO, USA), 1% HEPES (Sigma-Aldrich, St. Louis, MO, USA), and β-mercaptoethanol. The harvested cells were strained through a 70-µm strainer and were centrifuged (300× *g* for 5 min) and resuspended in ammonium chloride to lyse erythrocytes. For the leukocyte culture, cells were washed and resuspended in RPMI 1640 medium supplemented with 0.0001% GMCSF.

For the static adhesion assay, a 96-well plate was coated overnight with Mac-1 (10 µg/mL), CD147 (20 µg/mL), or BSA (1%). Hereafter, the coated wells were blocked with 4% BSA for 1 h. Then, 2 × 10^4^ isolated leukocytes from Mac-1^+/+^, Mac-1^−/−^, CD147^+/+^, or CD147^+/−^ mice were added into each well. After allowing the cells to adhere to the coating for one hour, the plate was washed twice with medium to remove non-adherent cells. The number of adherent cells was analyzed using photo documentation.

### 2.3. Isolation of Human Monocytes

Human monocytes were isolated as described before [26]. Briefly, mononuclear cells were isolated form venous blood drawn from the antecubital vein of healthy volunteers. The blood was collected in citrate-phosphate-dextrose-adenine (CPDA) buffer and was centrifuged over a Ficoll gradient at 920× *g* for 20 min. Leukocytes were cultured overnight in RPMI 1640 (Life technologies, Carlsbad, CA, USA) supplemented with 10% FCS (Life technologies, Carlsbad, CA, USA) and 1% Pen/Strep (Sigma-Aldrich, St. Louis, MO, USA) in plastic dishes. Non-adherent cells were removed by gentle washing. The adherent cells were detached using trypsin (Life technologies, Carlsbad, CA, USA) and were resuspended in RPMI 1640 supplemented with 10% FCS and 1% Pen/Strep for further use.

### 2.4. Isolation of Human Platelets

Human platelets were isolated as described before [27]. In brief, venous blood was drawn from the antecubital vein of healthy volunteers in adenosine citrate dextrose (ACD) buffer. The blood was centrifuged at 210× *g* for 20 min. Hereafter, the platelet-rich plasma (PRP) was collected and Tyrodes-HEPES buffer (HEPES 2.5 mM; NaCl, 150 mM; KCl, 1 mM; NaHCO_3_, 2.5 mM; glucose, 6 mM; BSA, 1 mg/mL; pH 7.4) was added and centrifuged at 836× *g* for 10 min. The pellet was resuspended in Tyrodes-HEPES buffer supplemented with 1 mM CaCl_2_ and 1 mM MgCl_2_ for further use.

### 2.5. Cell Adhesion under Flow Conditions

Using a flow chamber assay, human monocytes (2 × 10^5^/mL, stimulated with 1 µg/mL LPS (Sigma-Aldrich, St. Louis, MO, USA) for 2 h (n ≥ 7) or platelets (1 × 10^8^/mL, n = 7) were pre-incubated with anti-Mac-1 (clone #2LPM19c, monoclonal mouse-anti-Mac-1 Santa Cruz, Dallas, TX, USA), anti-CD147 antibody (clone #UM-8D6, monoclonal mouse-anti-CD147, Ancell, Bayport, MN, USA), or IgG control (all 20 µg/mL) for 2 h (monocytes) or 30 min (platelets). The cells were then perfused over Mac-1-, CD147-, or BSA-coated coverslips with arterial shear rates (2000 s^−1^). All experiments were recorded in real time and were analyzed for adherent and rolling monocytes/platelets, respectively.

### 2.6. Ethics Statement

All experiments were conducted in accordance with the German animal protection law and according to the Declaration of Helsinki. All protocols were conducted according to current animal protection laws and were approved by Regierungspräsidium Tübingen and the local ethics committee (Anzeige nach §8a TierSchG).

### 2.7. Statistical Analysis

Data are shown as mean ± SEM. Differences between groups with cardinal values were evaluated using Student’s *t*-test for two groups or ANOVA with subsequent post hoc analyses. Differences were considered statistically significant if *p* < 0.05. Analysis was performed using Prism 8 (GraphPad Software, La Jolla, CA, USA).

## 3. Results

### 3.1. Mac-1 Interaction with CD147 

**Figure 1:**
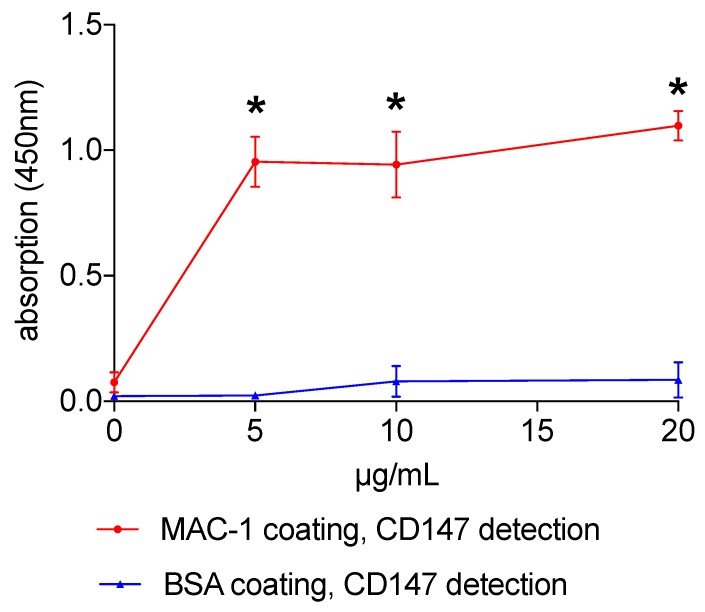
Mac-1 and CD147 interaction. Mac-1/CD147 interaction was assessed using an ELISA with recombinant Mac-1 or BSA (control) coating. Recombinant CD147 was added in ascending concentrations for 1 h. Evaluation was facilitated using a biotinylated secondary anti-CD147 antibody with a streptavidin/HRP complex. Changes in absorption of 3,3′,5,5′-tetramethylbenzidine were measured at 450 nm. Measurements from 6 independent experiments were analyzed, * indicates *p* < 0.05.

We used a binding ELISA to assess whether Mac-1 and CD147 can interact in a stable manner. As a negative control, coating with bovine serum albumin showed no significant interaction. When Mac-1 was used for coating the respective capture signal of CD147 was significantly enhanced, demonstrating a direct and stable interaction between the two molecules (Figure 1).

### 3.2. Mac-1/CD147 Interaction under Static Conditions

**Figure 2:**
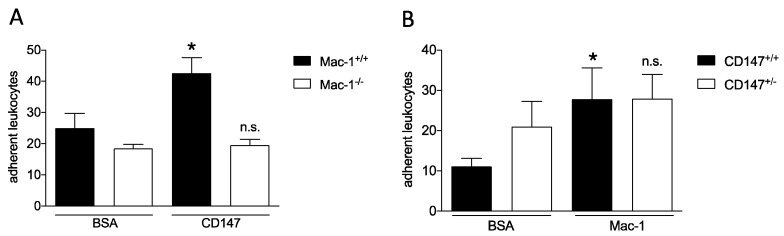
Mac-1/CD147 interaction in static leukocyte adhesion. A static adhesion assay with leukocytes form wild-type, Mac^−/−^ (n = 5) (**A**), and CD147^+/−^ (n = 12) (**B**) mice was used. Leukocytes were allowed to adhere to CD147- or BSA-coated surfaces (**A**) or to Mac-1- or BSA-coated surfaces (**B**) for 1 h. * indicates *p* < 0.05, n.s. indicates *p* > 0.05 compared to BSA control, respectively.

Based on the initial interaction study, we hypothesized that the interaction of these molecules could play a role in leukocyte adhesive events, as both have been described in this context separately. To this end, we performed a static adhesion assay with leukocytes from Mac-1-deficient and -sufficient mice. Mac-1^−/−^ leukocytes showed a markedly reduced adhesion to immobilized CD147 compared to wild-type mice, indicating a relevant binding strength. With heterozygous leukocytes from CD147^+/−^ mice, the effect was expected to be less pronounced. CD147^+/−^ cells showed a higher adhesion under basal conditions with a slight non-significant increase of adhesion to the Mac-1-coated surface (Figure 2).

### 3.3. Mac-1/CD147 Interaction under Dynamic Conditions

**Figure 3:**
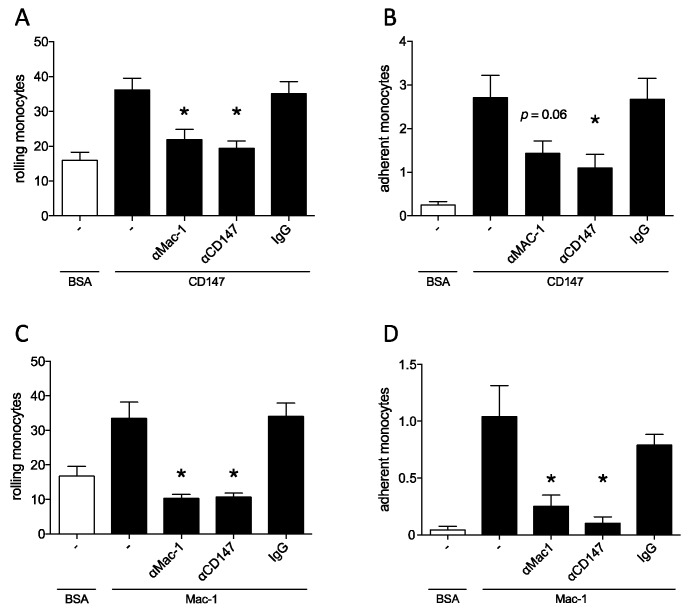
Mac-1/CD147 interaction facilitates leukocyte adhesion under dynamic flow conditions. To evaluate the relevance of the Mac-1/CD147 interaction for adhesion under arterial shear conditions, a flow chamber assay was used. LPS-stimulated human monocytes were perfused over BSA- or CD147-coated surfaces (**A** rolling cells, **B** adherent cells), or over BSA- or Mac-1-coated surfaces (**C** rolling cells, **D** adherent cells). Inhibitory antibodies against CD147 or Mac-1, as well as IgG control were added to determine the relevance of the respective protein for adhesion to its proposed counterpart. Results of ≥7 independent experiments are shown, * indicates *p* < 0.05 compared to uninhibited control.

After establishing a possibly relevant interaction strength for pathophysiological mechanisms, we studied LPS-stimulated human monocytes, as prototypical members of innate immunity under dynamic flow conditions. LPS-stimulated leukocytes were perfused over coated surfaces to evaluate the relative ability of the Mac-1/CD147 interaction to induce adhesion. Using a flow chamber assay, we observed a stable number of rolling and adherent cells on CD147- as well as on Mac-1-coated surfaces, compared to BSA under arterial shear rates (n = 9). In both cases, the effect could be reduced by the addition of either anti-CD147 or anti-Mac-1 antibody to the setup. The number of adherent monocytes over CD147 failed to reach statistical significance when treated with anti-Mac-1 (*p* = 0.06) (Figure 3).

### 3.4. Mac-1/CD147 Interaction in Platelets

**Figure 4:**
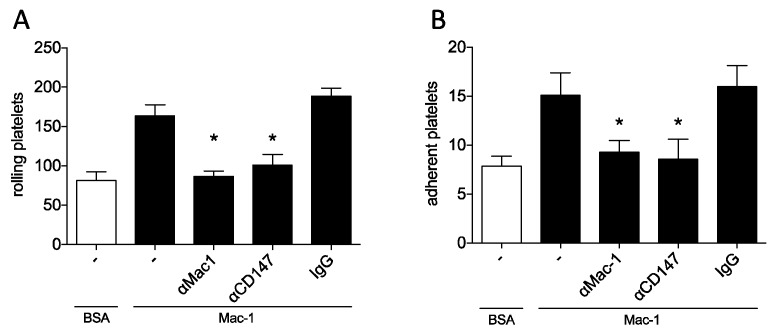
Mac-1/CD147 interaction facilitates platelet adhesion under dynamic flow conditions. The adhesion of platelets was studied in a similar flow chamber assay with arterial shear conditions. Isolated human platelets were perfused over Mac-1- or BSA-coated surfaces. Inhibiting antibodies against CD147 or Mac-1, as well as IgG control were added to determine the relevance of the protein for adhesion. Video analysis was performed to assess rolling (**A**) and adherent cells (**B**). Results of 7 independent experiment are shown, * indicates *p* < 0.05 compared to uninhibited control.

To establish whether this mechanism is specific to leukocytes, we decided to perform a similar dynamic adhesion assay using isolated human platelets. When isolated platelets were perfused over immobilized Mac-1, adhesion was greatly induced compared to perfusion over BSA, which was used as a control. When anti-Mac-1 or anti-CD147 antibody was added, adhesion to Mac-1 was significantly reduced, almost reaching control levels (n = 7). The addition of IgG antibody had no modifying effect (Figure 4).

## 4. Discussion

The recruitment of leukocytes is a vital mechanism for innate and adaptive immunological response. Especially, integrins have been found to play a central role for the adhesion of activated leukocytes to the endothelium [28].

CD147 is a promiscuous surface receptor expressed on a variety of cell types. Ligand binding to CD147 has been shown to elicit many pro-inflammatory and pro-fibrotic aspects, such as induction of matrix-metalloproteinases, induction of leukocyte chemotaxis, platelet activation, and adhesion [4,7,8,29].

Thus far, especially, homodimeric signaling of CD147 and the interaction with extracellular cyclophilin A has been characterized in the inflammatory and pro-fibrotic context [30,31]. In this study, we proposed Mac-1 as a previously unknown binding partner for CD147 on leukocytes and platelets.

Initial interaction studies showed a stable binding of the two molecules, making detection via an ELISA-based interaction assay feasible. As both molecules have been found to be involved in leukocyte recruitment, we used static and dynamic adhesion assays to establish whether the stability of the interaction could be of pathophysiological significance. We found that inhibition of either binding partner strongly reduced the ability of leukocytes and especially monocytes to adhere to CD147- or Mac-1-coated surfaces under static and dynamic conditions.

With this in mind, we were interested in identifying the mechanism by using leukocytes from Mac-1 knock-out mice and CD147 heterozygous mice. Both genotypes showed little adhesion to the immobilized binding partner, whereas leukocytes from wild-type mice showed strong adhesion. Interestingly, this interaction also seems to be of importance for the adhesion of platelets. While platelets play a role in inflammation by releasing pro-inflammatory and pro-fibrotic chemokines, in clinical practice, their pro-thrombotic properties are especially of great interest, as the initial contact of the platelets with the activated endothelium and associated factors triggers a wide range of responses, including degranulation, activation by the presentation of adhesion molecules, and shape changes. Under flow conditions, platelet adhesion to Mac-1 was abolished by the addition of anti-Mac-1 or anti-CD147 antibodies. This finding suggests that the binding between Mac-1 and CD147 may mediate platelet–leukocyte interactions as well.

CD147 has been identified to play a prominent role in the induction of pro-inflammatory and pro-thrombotic effects in various disease models and across species. Our group has previously described platelet glycoprotein VI (GPVI) as a novel receptor for CD147, mediating platelet rolling and monocyte–platelet aggregates [11]. Furthermore, the interaction of CD147 with cyclophilin A (CyPA) has been intensively investigated in the context of thromboinflammation. CyPA can be released from the cytoplasm by various pro-inflammatory stimuli. The binding of CyPA to CD147 on the surface of platelets induces rapid degranulation and activation, leading to shape changes and the release of pro-inflammatory chemokines [4]. Furthermore, the CD147–CyPA interaction induces leukocyte chemotaxis and activation, expression of matrix-metalloproteinases, promotes atherosclerotic plaque formation, and plays critical roles in several inflammatory and cardiovascular diseases [14,31,32,33]. CD147 has also been associated with other integrins, including α_3_β_1_ and α_6_β_1_ [2]. S100A9, belonging to the S100 family of proteins, has been identified to induce leukocyte migration, thrombosis, and cytokine release via CD147 [5,34]. Mac-1, predominantly expressed on myeloid cells, has also been extensively implicated in various thromboinflammatory mechanisms, including leukocyte migration, phagocytosis, and the facilitation of monocyte–platelet aggregates [15,35].

The presented data therefore links two molecules, both deeply embedded in thromboinflammatory mechanisms, complimenting the existing literature. However, the pathophysiological importance in vivo remains to be elucidated. This study was primarily designed to establish a plausible link between CD147 and Mac-1 in the context of thromboinflammation using in vitro experiments. Further investigations are necessary to characterize the underlying signaling mechanisms and to subsequently evaluate its pathophysiological relevance.

## 5. Conclusions

In this study, we described for the first time the interaction of integrin Mac-1 with CD147. We furthermore provide evidence that CD147 is a novel and relevant binding partner for Mac-1 on leukocytes and platelets.

## References

[1] Pircher J., Engelmann B., Massberg S., Schulz C. (2019). Platelet-neutrophil crosstalk in atherothrombosis. Thromb. Haemost..

[2] Berditchevski F., Chang S., Bodorova J., Hemler M.E. (1997). Generation of monoclonal antibodies to integrin-associated proteins. Evidence that alpha3beta1 complexes with EMMPRIN/basigin/OX47/M6. J. Biol. Chem..

[3] Dai J.Y., Dou K.F., Wang C.H., Zhao P., Lau W.B., Tao L., Wu Y.M., Tang J., Jiang J.L., Chen Z.N. (2009). The interaction of HAb18G/CD147 with integrin alpha6beta1 and its implications for the invasion potential of human hepatoma cells. BMC Cancer.

[4] Seizer P., Ungern-Sternberg S.N., Schonberger T., Borst O., Munzer P., Schmidt E.M., Mack A.F., Heinzmann D., Chatterjee M., Langer H. (2015). Extracellular cyclophilin A activates platelets via EMMPRIN (CD147) and PI3K/Akt signaling, which promotes platelet adhesion and thrombus formation in vitro and in vivo. Arterioscler. Thromb. Vasc. Biol..

[5] Hibino T., Sakaguchi M., Miyamoto S., Yamamoto M., Motoyama A., Hosoi J., Shimokata T., Ito T., Tsuboi R., Huh N.-H. (2013). S100A9 is a novel ligand of EMMPRIN that promotes melanoma metastasis. Cancer Res..

[6] Muramatsu T. (2016). Basigin (CD147), a multifunctional transmembrane glycoprotein with various binding partners. J Biochem..

[7] Seizer P., Ochmann C., Schonberger T., Zach S., Rose M., Borst O., Klingel K., Kandolf R., MacDonald H.R., Nowak R.A. (2011). Disrupting the EMMPRIN (CD147)-cyclophilin A interaction reduces infarct size and preserves systolic function after myocardial ischemia and reperfusion. Arterioscler. Thromb. Vasc. Biol..

[8] Seizer P., Schonberger T., Schott M., Lang M.R., Langer H.F., Bigalke B., Kramer B.F., Borst O., Daub K., Heidenreich O. (2010). EMMPRIN and its ligand cyclophilin A regulate MT1-MMP, MMP-9 and M-CSF during foam cell formation. Atherosclerosis.

[9] Elvers M., Herrmann A., Seizer P., Munzer P., Beck S., Schonberger T., Borst O., Martin-Romero F.J., Lang F., May A.E. (2012). Intracellular cyclophilin A is an important Ca^2+^ regulator in platelets and critically involved in arterial thrombus formation. Blood.

[10] Seizer P., Geisler T., Bigalke B., Schneider M., Klingel K., Kandolf R., Stellos K., Schreieck J., Gawaz M., May A.E. (2013). EMMPRIN and its ligand Cyclophilin A as novel diagnostic markers in inflammatory cardiomyopathy. Int. J. Cardiol..

[11] Seizer P., Borst O., Langer H.F., Bultmann A., Munch G., Herouy Y., Stellos K., Kramer B., Bigalke B., Buchele B. (2009). EMMPRIN (CD147) is a novel receptor for platelet GPVI and mediates platelet rolling via GPVI-EMMPRIN interaction. Thromb. Haemost..

[12] Flora G.K., Anderton R.S., Meloni B.P., Guillemin G.J., Knuckey N.W., MacDougall G., Matthews V., Boulos S. (2019). Microglia are both a source and target of extracellular cyclophilin A. Heliyon.

[13] von Ungern-Sternberg S.N.I., Vogel S., Walker-Allgaier B., Geue S., Maurer A., Wild A.M., Munzer P., Chatterjee M., Heinzmann D., Kremmer E. (2017). Extracellular cyclophilin A augments platelet-dependent thrombosis and thromboinflammation. Thromb. Haemost..

[14] von Ungern-Sternberg S.N.I., Zernecke A., Seizer P. (2018). Extracellular matrix metalloproteinase inducer EMMPRIN (CD147) in cardiovascular disease. Int. J. Mol. Sci..

[15] Bednarczyk M., Stege H., Grabbe S., Bros M. (2020). β2 Integrins-multi-functional leukocyte receptors in health and disease. Int. J. Mol. Sci..

[16] Futosi K., Fodor S., Mocsai A. (2013). Neutrophil cell surface receptors and their intracellular signal transduction pathways. Int. Immunopharmacol..

[17] Mitroulis I., Alexaki V.I., Kourtzelis I., Ziogas A., Hajishengallis G., Chavakis T. (2015). Leukocyte integrins: Role in leukocyte recruitment and as therapeutic targets in inflammatory disease. Pharmacol. Ther..

[18] Keiper T., Al-Fakhri N., Chavakis E., Athanasopoulos A.N., Isermann B., Herzog S., Bohle R.M., Haendeler J., Preissner K.T., Santoso S. (2005). The role of junctional adhesion molecule-C (JAM-C) in oxidized LDL-mediated leukocyte recruitment. FASEB J. Off. Publ. Fed. Am. Soc. Exp. Biol..

[19] Chavakis T., Athanasopoulos A., Rhee J.S., Orlova V., Schmidt-Woll T., Bierhaus A., May A.E., Celik I., Nawroth P.P., Preissner K.T. (2005). Angiostatin is a novel anti-inflammatory factor by inhibiting leukocyte recruitment. Blood.

[20] Hajishengallis G., Chavakis T. (2013). Endogenous modulators of inflammatory cell recruitment. Trends Immunol..

[21] Li N., Yang H., Wang M., Lu S., Zhang Y., Long M. (2018). Ligand-specific binding forces of LFA-1 and Mac-1 in neutrophil adhesion and crawling. Mol. Biol. Cell.

[22] Chavakis T., Santoso S., Clemetson K.J., Sachs U.J., Isordia-Salas I., Pixley R.A., Nawroth P.P., Colman R.W., Preissner K.T. (2003). High molecular weight kininogen regulates platelet-leukocyte interactions by bridging Mac-1 and glycoprotein Ib. J. Biol. Chem..

[23] Santoso S., Sachs U.J., Kroll H., Linder M., Ruf A., Preissner K.T., Chavakis T. (2002). The junctional adhesion molecule 3 (JAM-3) on human platelets is a counterreceptor for the leukocyte integrin Mac-1. J. Exp. Med..

[24] Lishko V.K., Podolnikova N.P., Yakubenko V.P., Yakovlev S., Medved L., Yadav S.P., Ugarova T.P. (2004). Multiple binding sites in fibrinogen for integrin alphaMbeta2 (Mac-1). J. Biol. Chem..

[25] Vorup-Jensen T., Jensen R.K. (2018). Structural immunology of complement receptors 3 and 4. Front. Immunol..

[26] Schmidt R., Bultmann A., Ungerer M., Joghetaei N., Bulbul O., Thieme S., Chavakis T., Toole B.P., Gawaz M., Schömig A. (2006). Extracellular matrix metalloproteinase inducer regulates matrix metalloproteinase activity in cardiovascular cells: Implications in acute myocardial infarction. Circulation.

[27] Langer H.F., Stellos K., Steingen C., Froihofer A., Schonberger T., Kramer B., Bigalke B., May A.E., Seizer P., Muller I. (2009). Platelet derived bFGF mediates vascular integrative mechanisms of mesenchymal stem cells in vitro. J. Mol. Cell. Cardiol..

[28] Kourtzelis I., Mitroulis I., von Renesse J., Hajishengallis G., Chavakis T. (2017). From leukocyte recruitment to resolution of inflammation: The cardinal role of integrins. J. Leukoc. Biol..

[29] Yuan W., Ge H., He B. (2010). Pro-inflammatory activities induced by CyPA-EMMPRIN interaction in monocytes. Atherosclerosis.

[30] Yoshida S., Shibata M., Yamamoto S., Hagihara M., Asai N., Takahashi M., Mizutani S., Muramatsu T., Kadomatsu K. (2000). Homo-oligomer formation by basigin, an immunoglobulin superfamily member, via its N-terminal immunoglobulin domain. Eur. J. Biochem. FEBS.

[31] Seizer P., Gawaz M., May A.E. (2014). Cyclophilin A and EMMPRIN (CD147) in cardiovascular diseases. Cardiovasc. Res..

[32] Xu Q., Leiva M.C., Fischkoff S.A., Handschumacher R.E., Lyttle C.R. (1992). Leukocyte chemotactic activity of cyclophilin. J. Biol. Chem..

[33] Kim H., Kim W.J., Jeon S.T., Koh E.M., Cha H.S., Ahn K.S., Lee W.H. (2005). Cyclophilin A may contribute to the inflammatory processes in rheumatoid arthritis through induction of matrix degrading enzymes and inflammatory cytokines from macrophages. Clin. Immunol..

[34] Alexaki V.I., May A.E., Fujii C., V Ungern-Sternberg S.N., Mund C., Gawaz M., Chavakis T., Seizer P. (2017). S100A9 induces monocyte/macrophage migration via EMMPRIN. Thromb. Haemost..

[35] Kourtzelis I., Kotlabova K., Lim J.H., Mitroulis I., Ferreira A., Chen L.S., Gercken B., Steffen A., Kemter E., Klotzsche-von Ameln A. (2016). Developmental endothelial locus-1 modulates platelet-monocyte interactions and instant blood-mediated inflammatory reaction in islet transplantation. Thromb. Haemost..

